# High-resolution 3D light-field imaging

**DOI:** 10.1117/1.JBO.25.10.106502

**Published:** 2020-10-12

**Authors:** Qiang Geng, Zhiqiang Fu, Shih-Chi Chen

**Affiliations:** The Chinese University of Hong Kong, Department of Mechanical and Automation Engineering, Shatin, Hong Kong, China

**Keywords:** fluorescence imaging, light-field microscopy, image deconvolution, phase retrieval

## Abstract

**Significance:** High-speed 3D imaging methods have been playing crucial roles in many biological discoveries.

**Aim:** We present a hybrid light-field imaging system and image processing algorithm that can visualize high-speed biological events.

**Approach:** The hybrid light-field imaging system uses the selective plane optical illumination, which simultaneously records a high-resolution 2D image and a low-resolution 4D light-field image. The high-resolution 4D light-field image is obtained by applying the hybrid algorithm derived from the deconvolution and phase retrieval methods.

**Results:** High-resolution 3D imaging at a speed of 100-s volumes per second over an imaging field of $250\times 250\times 80\;{\mu m}^{3}$ in the x, y, and z axis, respectively, is achieved with a 2.5 times enhancement in lateral resolution over the entire imaging field compared with standard light-field systems. In comparison to the deconvolution algorithm, the hybrid algorithm addresses the artifact issue at the focal plane and reduces the computation time by a factor of 4.

**Conclusions:** The new hybrid light-field imaging method realizes high-resolution and ultrafast 3D imaging with a compact setup and simple algorithm, which may help discover important applications in biophotonics to visualize high-speed biological events.

## 1 Introduction

Visualization of 3D high-speed biological events, e.g., signaling of neural circuits, has been the driving force of many emerging 3D imaging techniques, such as random-access microscopy^1^^,^^2^ and light-sheet microscopy (LSM).^3^^,^^4^ The former approach selectively scans the laser beam in the regions of interest to monitor biological events at speeds up to 10-s kHz,^1^^,^^2^ and the latter approach exploits a light sheet to parallelly illuminate and record 2D images at frame rates up to 100-s Hz;^3^^,^^4^ accordingly, clear 3D images can be obtained through the application of custom-designed phase masks^3^ or synchronization with an axial scanner,^4^ achieving a volume image rate of ∼30  Hz.

Light-field microscopy (LFM)^5^ is a high-speed 3D imaging technique that captures a volume image via a single exposure from a camera, where the imaging speed is limited by cameras. Briefly, a 3D image in LFM can be restored from a recorded “light-field image” that simultaneously contains the positions of objects, i.e., 2D image, and angles of illumination, i.e., another 2D image, which is termed a “4D” image. Comparing with other fast fluorescent imaging methods, LFM achieves the highest volume imaging rate at the expense of low spatial and angular resolution,^5^^,^^6^ which prevents the broad adoption of LFM in biological studies. To address the issue, both computational and optical methods have been developed. For example, deconvolution algorithms have been applied to LFM^7^ for imaging neuronal activities at high speed.^8^^–^^10^ Although simple and effective, this method is prone to generating artifacts near the native image plane (NIP) and requires powerful computers for processing dense 3D images. In addition to the deconvolution algorithm, the phase space retrieval method^11^ has been developed to computationally recover a high-resolution light-field image. Comparing with the deconvolution method, this method shows decreasing fidelity away from the NIP. In terms of optical methods, insertion of phase masks,^12^ optimization of optical distances,^13^ adoption of an orthogonal detection arm,^14^ and selective plane illumination^15^ have been demonstrated to improve the spatial and angular resolution of LFM. However, the increased complexity in optical design often prevents the easy application of computational methods, e.g., deconvolution algorithms.

In this work, we present a new hybrid LFM via selective optical illumination, which reconstructs a high-resolution 4D light-field image from a simultaneously recorded 2D image and low-resolution 4D light-field image. Briefly, multi-layer 2D images within the depth of the focus (DOF) are generated via deconvolution. Next, the multi-layer images are used as constraints for the iterated Fourier transform of the interpolated 4D light-field image to reconstruct a high-resolution 4D light-field image. Accordingly, the hybrid LFM achieves an imaging speed of 100-s volumes per second (vps) with a 3D field of view of $250\times 250\times 80\;{\mu m}^{3}$ in the x,y, and z axes, respectively. The measured axial and lateral resolutions, i.e., full width at half maximum (FWHM), are 9.36 and 1.85  μm, respectively.

## 2 Experiments

### 2.1 Optical Configuration of the Hybrid LFM

**Fig. 1:**
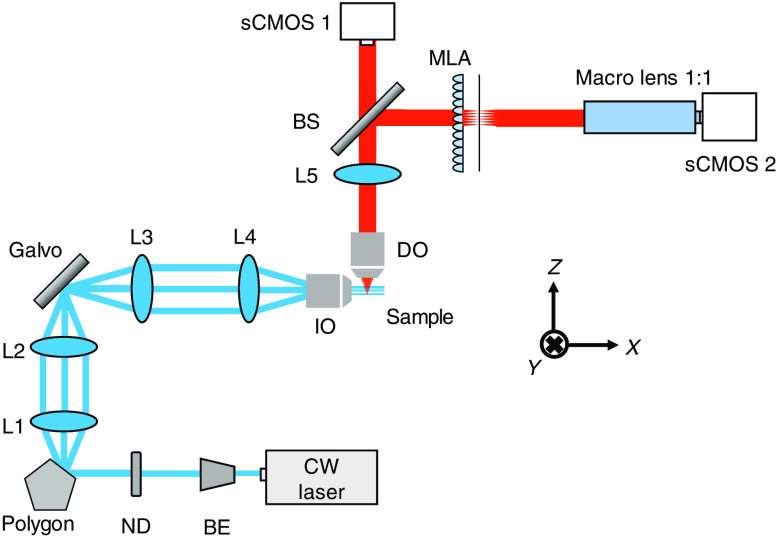
Optical configuration of the hybrid LFM.

Figure 1 shows the optical configuration of the hybrid LFM. The light source is a 488-nm continuous-wave laser (MLD, Cobolt). The laser first passes through the beam expander and neutral density filter and enters the polygonal mirror scanner (DT-72-290-025, Lincoln Laser), which scans the laser beam horizontally to form a light sheet. Next, the light sheet is relayed to a galvanometric scanner (6220HB, Cambridge Technology) via a 4-f system, i.e., L1 and L2, and scanned vertically over a range of ±40  μm. Then, the laser is coupled to the illumination objective (Plan 10×/0.30  W, Nikon), which images the scanning light sheet to the focal region to form a uniformly illuminated imaging volume of $250\times 250\times 80\;{\mu m}^{3}$. For detection, the emissions are first collected by the detection objective (DO) (APO LWD 40×/0.80  W, Nikon) and coupled to two imaging arms via a tube lens (L5). The emissions are equally separated by a beam splitter (50:50, Chroma) to a scientific complementary metal-oxide-semiconductor (sCMOS) camera, i.e., sCMOS 1 (Zyla 5.5, Andor), which generates high-resolution 2D images; and a light-field camera, which consists of a microlens array (MLA, APO-Q-P150-F3.5, OKO Tech), macrolens (180-mm F3.5, Canon), and sCMOS 2 (C13440, Hamamatsu). Specifically, the MLA is located at the focal plane of L5, and the macrolens maps the MLA focal plane to sCMOS 2. (It is worthy to note that the two sCMOS cameras have the same pixel sizes of 6.5  μm.) Lastly, the collected 2D and light-field images are combined to form high-resolution light-field images via a custom-developed program.

**Fig. 2:**
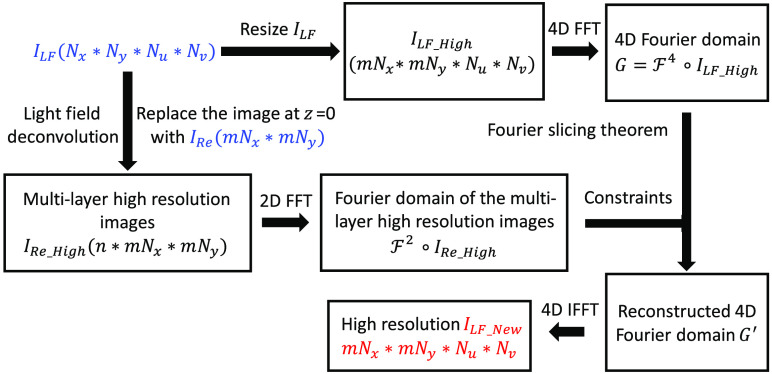
Flowchart of the hybrid algorithm.

Figure 2 shows the flowchart of the hybrid algorithm. $I_{\mathrm{LF}}$ ($N_{x}*N_{y}*N_{u}*N_{v}$) and $I_{\text{Re}}$ ($mN_{x}*mN_{y}$) represents low-resolution light-field images from sCMOS 2 and high-resolution 2D images from sCMOS 1, respectively, where (x,y) and (u,v) represent the position and angular coordinates; and m is an interpolation constant. $I_{\text{Re}}$ and $I_{\mathrm{LF}}$ are simultaneously obtained by scanning the light sheet vertically over a range of ±40  μm via galvo within a single exposure time of sCMOS cameras, i.e., 10 ms. To begin,$I_{\mathrm{LF}}$ is resized to $I_{\mathrm{LF}\_\mathrm{High}}$ by a factor of m in the spatial domain via the bicubic interpolation, followed by a 4D fast Fourier transform (FFT) to obtain G, i.e., 4D Fourier information. It is worthy to note that $F^{n}$ and $F^{-n}$ represent the n-dimension FFT and inverse FFT (IFFT), respectively. Next, multidepth high-resolution images IRe_High ($n*mN_{x}*mN_{y}$) (n=number of layers) are calculated from $I_{\mathrm{LF}}$ via deconvolution, where the image at the focal plane (z=0) is replaced by the high-resolution image $I_{\text{Re}}$. It is worthy to note that based on Fourier slicing theorem FFT of IRe_High represents slices along different directions in the Fourier domain of the 4D light-field data, which is used as constraints for slicing in the 4D Fourier domain G. Lastly, a 4D IFFT is performed to reconstruct the 4D Fourier signals $G^{\prime }$ in the spatial domain. By setting the negative and complex numbers to zero, high-resolution light field images, i.e., $I_{\mathrm{LF}\_\mathrm{new}}$ ($mN_{x}*mN_{y}*N_{u}*N_{v}$), are obtained. Table 1 presents the pseudocodes of the hybrid algorithm.

**Table 1:** Pseudocode of hybrid LFM algorithm.

1: Obtain $I_{\text{Re}}$ and $I_{\mathrm{LF}}$ from sCMOS 1 and sCMOS 2, simultaneously
2: Calculate $I_{\mathrm{LF}\_\mathrm{High}}$ by resizing $I_{\mathrm{LF}}$ with a magnification of m
3: Obtain the 4D Fourier domain G of $I_{\mathrm{LF}\_\mathrm{High}}$ by 4D FFT
4: Calculate IRe⁡_High by deconvolution from $I_{\mathrm{LF}}$
5: // the image at the focal plane is replaced by $I_{\text{Re}}$
6: Obtain the 2D Fourier domain $G_{f}^{\prime }$ of IRe⁡_High by 2D FFT
7: // $G_{f}^{\prime }$ is used as constraints in the Fourier slicing theorem
8: For each discrete image depth f, do
9: Calculate the depth parameter α=f/F; (*F* = MLA focal length)
10: Update the 4D Fourier space by slicing at f by:
11: $$G[x^{\prime },y^{\prime },\frac{x^{\prime }}{mN_{x}}(\alpha -1),\frac{y^{\prime }}{mN_{y}}(\alpha -1)]=G_{f}^{\prime }(x^{\prime },y^{\prime });$$
12: end
13: Obtain 4D high resolution image $I_{\mathrm{LF}\_\mathrm{new}}$ from G by 4D IFFT

### 2.2 Experimental Results

**Fig. 3:**
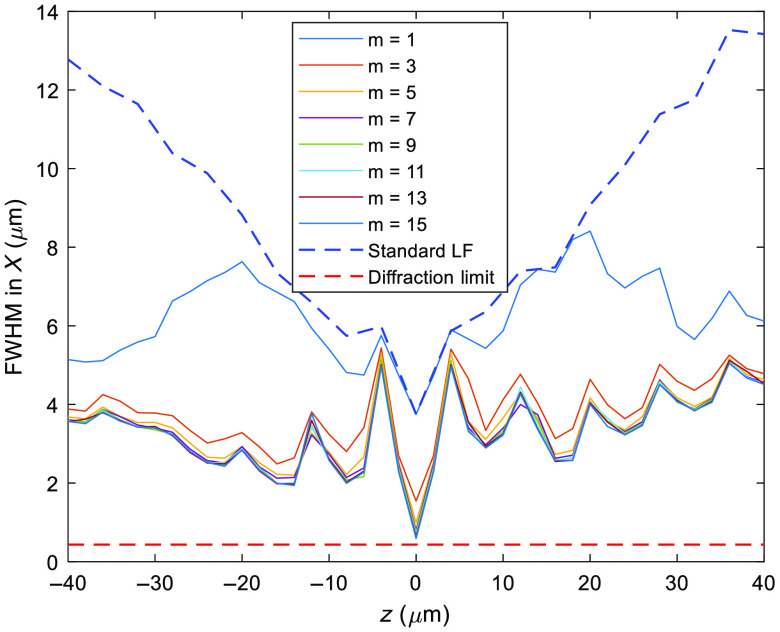
Lateral resolution of the hybrid LFM under various m values along the z axis.

This section presents the experimental results of the proposed hybrid LFM. First, we discuss the resolution enhancement over the standard LFM. For standard LFM, the resolution can be estimated by the size of a microlens ($d_{\text{pitch}}$) and the magnification (M) of the DO, i.e., resolution $R_{\text{lateral}}=d_{\text{pitch}}/M=150\;\mu m/40=3.75\;\mu m$ at the focal plane (z=0). It is worthy to note that the lateral resolution decreases when the imaging plane moves away from the focal plane. For the hybrid algorithm, a large m value can directly improve the lateral resolution of the reconstructed images, especially at the focal plane. As m continues to increase, the resolution enhancement effect gradually diminishes; in the meantime, the required computational power increases at a rate of $m^{2}$. To select the optimal value of m, simulated experiments have been devised and conducted in MATLAB based on wave optics and scalar Debye theory.^7^^,^^16^ In the simulation, we calculate the fluorescence imaging results from sCMOS 1 and sCMOS 2, where the emissions are from an infinitely small spot at the focal plane. Next, the two simulated images are imported to the hybrid algorithm. Specifically, 11 2D images from z=−40 to 40  μm with 8  μm intervals are generated via deconvolution, where the image at the focal plane is replaced by the 2D image from sCMOS 1. The 11 high-resolution images IRe_High are used as constraints in the Fourier slicing theorem to reconstruct a high-resolution 4D light field image. As shown in Fig. 3, the lateral resolution of the reconstructed images is plotted as a function of the interpolation parameter m and axial positions; and as a reference, the resolution of the standard LFM and diffraction limited are also plotted together in Fig. 3. From the results, one may observe the lateral resolution has been improved over the entire scanning range and the effect becomes more prominent with increasing distances away from the focal plane, i.e., the resolution of the hybrid LFM remains relatively constant over the range in comparison with standard LFM. The resolution in the z axis fluctuates as the hybrid algorithm uses high-resolution deconvoluted images at the selected depths (i.e., z=±40, ±32, ±24, ±16, and ±8  μm) as constraints to reconstruct the entire 3D volume based on phase retrieval. As such, better resolution is observed at these positions. From the results, we can confirm the increasing m values will enhance the resolution with a diminishing effect for m larger than 5. Considering the tradeoffs between resolution and computational cost, we select m=3 in the hybrid algorithm for imaging experiments in the later sections; this is close to the resolution enhancement limit of the deconvolution algorithm for standard LFM.^7^

In terms of axial resolution, the hybrid LFM should have comparable resolution with standard LFM. To estimate the axial resolution, we first apply the Sparrow criterion,^17^ where the Sparrow limit can be described as the closest distance on the microlens imaging plane that two focal points can be resolved ($R_{\text{obj}}$):

$$R_{\mathrm{obj}}=\frac{0.47\lambda }{\mathrm{NA}}M,$$ (1)

where λ is the wavelength and NA and M are the numerical aperture and magnification of the DO, respectively. The total resolvable spots (N) per microlens can be determined by the size of individual microlenses ($d_{\text{pitch}}$), as described in Eq. (2)

$$N=\frac{d_{\mathrm{pitch}}}{R_{\mathrm{obj}}}.$$ (2)

Given $d_{\text{pitch}}=150\;\mu m$, M=40, and NA=0.8, from Eqs. (1) and (2), we can find $R_{\text{obj}}=12.5\;\mu m$ and N=12. From a previous study,^5^ the axial resolution ($R_{\text{axial}}$) and maximum axial imaging range ($z_{\text{range}}$) of LFM can be calculated via Eqs. (3) and (4), respectively,

$$R_{\mathrm{axial}}\approx \frac{(2+N)\lambda n}{2{\mathrm{NA}}^{2}},$$ (3)

$$z_{\mathrm{range}}\approx \frac{(2+N^{2})\lambda n}{2{\mathrm{NA}}^{2}},$$ (4)

where N represents the resolvable spots in a microlens, and n is the refractive index of immersion media (n=1.33 for water). By substituting our system parameters into Eqs. (3) and (4), the axial resolution ($R_{\text{axial}}$) and imaging range ($z_{\text{range}}$) of the hybrid LFM are found to be 7.74 and 80.24  μm, respectively. It is worthy to note that within the working volume, i.e., −40 to 40  μm, the axial resolution decreases as the imaging plane moves away from the focus.

**Fig. 4:**
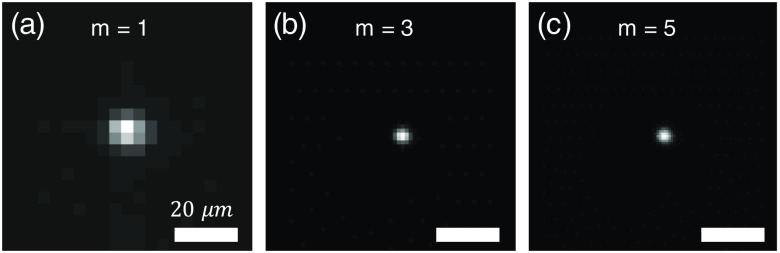
Reconstructed lateral PSF at z=32  μm for different m values.

To verify the predicted resolution and range, we performed imaging experiments on 1-μm fluorescent beads (F8819, Thermo Fisher Scientific) at different depths to characterize the lateral and axial point spread functions (PSF) throughout the entire imaging volume. The recorded raw images are processed by the hybrid LFM algorithm. To begin, we first identify the optimal value of m by setting m to different values and recording the corresponding images at z=32  μm, i.e., an out-of-focus position. The results are shown in Figs. 4(a)–4(c), where m is set to 1, 3, and 5, respectively. From the results, one may observe that the lateral resolution is improved substantially when m is equal or >3. When m=5, the enhancement becomes visually indistinguishable, which is consistent with our prediction, as shown in Fig. 3.

**Fig. 5:**
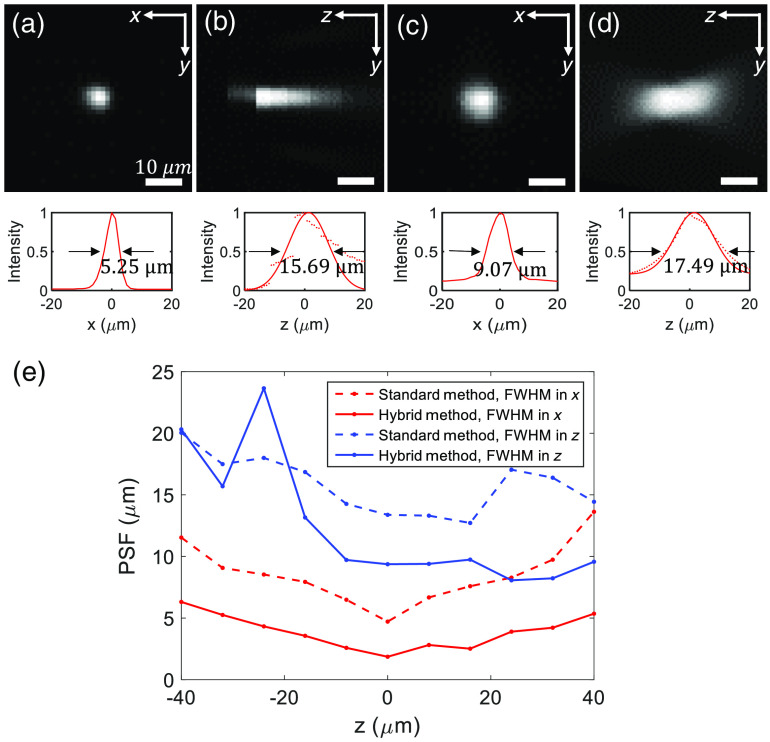
3D PSF of the hybrid and standard LFM.

We record the lateral and axial PSF images over the entire work volume, followed by image reconstruction (m=3) via the hybrid algorithm. Figures 5(a) and 5(b) show the reconstructed lateral and axial PSF, i.e., 5.25 and 15.69  μm, respectively, of the hybrid LFM at z=−32  μm, i.e., worst-case scenario at the far end of the imaging field. In comparison, Figs. 5(c) and 5(d) show the lateral and axial PSF, i.e., 9.07 and 17.49  μm, respectively, of the standard LFM at z=−32  μm. Figure 5(e) plots the reconstructed lateral and axial PSF over the entire volume for both the hybrid and standard LFM. From the results, we can conclude the hybrid FLM has effectively improved the lateral resolution by a factor of ∼2.5 over the entire imaging field. The lateral and axial PSF measured at the focal plane (z=0) are 1.85 and 9.36  μm, respectively. We also find the hybrid LFM achieves better axial resolution than the standard LFM in the vicinity of the focal region, i.e., z=±15  μm.

**Fig. 6:**
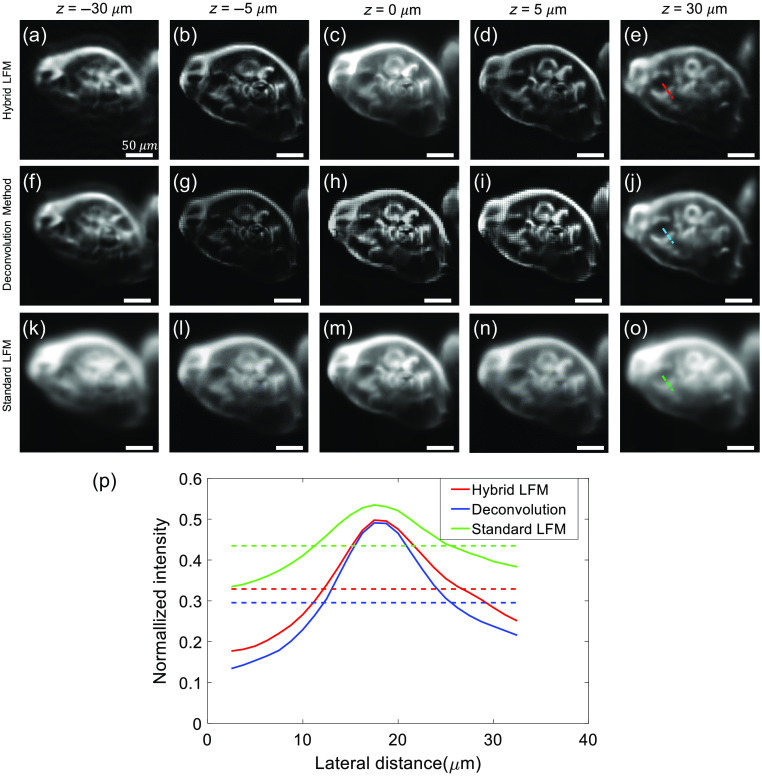
3D image of fern spores reconstructed via the hybrid algorithm, standard LFM, and deconvolution algorithm. (a)–(e) Reconstructed cross-sectional images from the hybrid algorithm at z=−30, −5, 0, 5, and 30  μm, respectively; (f)–(j) reconstructed images from the deconvolution algorithm at z=−30, −5, 0, 5, and 30  μm, respectively; (k)–(o) reconstructed images from the standard LFM at z=−30, −5, 0, 5, and 30  μm, respectively; and (p) normalized intensity profiles along the red, blue, and green dashed lines in (e), (j), and (o), respectively. Scale bar=50  μm. (See Videos 1 and 2 for demonstration.) (Video 1 [URL: https://doi.org/10.1117/1.JBO.25.10.106502.1] MP4, 303 KB and Video 2 [URL: https://doi.org/10.1117/1.JBO.25.10.106502.2] MP4, 176 KB).

In this section, we perform 3D imaging experiments on biological specimens, i.e., fern spores (B17124 R8, Walter Products Inc.), to verify the performance of our hybrid LFM. Figures 6(a)–6(e) show reconstructed images from the hybrid LFM at five different depths, i.e., −30, −5, 0, 5, and 30  μm, with a field of view of $250\times 250\;{\mu m}^{2}$. (It is worthy to note that z=0  μm corresponds to the DO focal plane). In comparison, Figs. 6(f)–6(j) show reconstructed images from the deconvolution algorithm; and Figs. 6(k)–6(o) show images obtained from a standard LFM at the same imaging field. From the results, one may find the hybrid system yields images of better resolution and contrast throughout the imaging field in comparison to the standard LFM. Comparing with the deconvolution method, we find that the hybrid algorithm shows improved resolution around the focal plane, i.e., z=±5  μm, without any artifact; and comparable resolution at regions away from the focal plane (z=±30  μm). In terms of computation time, the hybrid algorithm can improve the speed by a factor of 3 to 4 due to the use of sparse deconvolution images, e.g., 10 deconvoluted images in our hybrid imaging process in Fig. 6. For example, when processing a light-field image with a size of 1892×1892  pixels, the deconvolution algorithm uses 56 min to reconstruct a 3D image stack from −40 to 40  μm with a step of 2  μm; in contrast, the hybrid algorithm uses 16 min to reconstruct a 3D image stack over the same imaging field. These calculations are performed on a workstation equipped with Intel(R) Core (TM) i9-7920X CPU at 2.90 GHz, 64 G RAM, and Nvidia Quadro P4000 graphic cards. To compare quantitatively, Fig. 6(p) plots the cross-sectional intensity profiles along the red, blue, and green dashed lines in Figs. 6(e), 6(j), and 6(o), respectively, where the intensity curves are normalized over each image, i.e., Figs. 6(e), 6(j), and 6(o), for fair comparisons. From the results, the calculated signal-to-background ratio (SBR) in Figs. 6(e), 6(j), and 6(o) are 1.55, 1.66, and 1.23 with a mean background signal of 0.32, 0.30, and 0.43, respectively. The imaging results affirm the performance improvement of the hybrid LFM over standard LFM and the deconvolution algorithm (in terms of speed and the resolution at the NIP). It is worthwhile to note that the imaging resolution of the hybrid LFM can be further increased using more than one light-sheet images, where each additional light-sheet image can improve the resolution over a local volume at the expense of increased system complexity and cost.

## 3 Conclusion

In conclusion, we have presented the design and characterization of a new hybrid LFM that generates high-resolution 3D images at a speed of 100-s vps over an imaging field of $250\times 250\times 80\;{\mu m}^{3}$ in the x,y, and z axis, respectively. The collected raw 2D and 4D light-field images are processed by the hybrid algorithm, developed based on the Fourier slicing theorem; to balance the resolution and computational power, an optimal interpolation constant (m) is selected experimentally. Analytical models have been developed to guide the system design and predict the performance, i.e., imaging resolution and range. In the experiments, we first characterize the 3D PSF of both the hybrid and standard LFM. The results confirm the lateral resolution has been improved for 2.5 times over the entire imaging field comparing with standard LFM. Next, 3D imaging experiments on fern spore samples have been performed, where the hybrid LFM demonstrates substantially improved resolution and SBR over the standard LFM. In comparison with the deconvolution algorithm, the hybrid LFM has addressed the artifact issue at the focal plane and reduced the computation time by a factor of 4. The improved resolution and speed present great opportunities for scientists to adopt LFM for *in vivo* biological studies.
